# Impact of acute inflammation on spinal motoneuron synaptic plasticity following ventral root avulsion

**DOI:** 10.1186/1742-2094-7-29

**Published:** 2010-05-04

**Authors:** Roberta Barbizan, Alexandre LR Oliveira

**Affiliations:** 1 Department of Anatomy, Cell Biology, Physiology and Biophysics, Institute of Biology, University of Campinas (UNICAMP), CP 6109, CEP 13083-970, Campinas, SP, Brazil

## Abstract

**Background:**

Ventral root avulsion is a proximal nerve root lesion in which ventral motor nerve rootlets are torn from surface of the spinal cord, resulting in extensive death of motoneurons. It has been previously shown that if such lesioning is performed in an animal with experimental autoimmune encephalomyelitis (EAE), a significant number of motoneurons can be rescued despite an intense inflammatory reaction. This rescue effect has been attributed to production of a number of neurotrophic factors by invading T cells. Synaptological changes may be involved in neuronal degeneration, and a better understanding of the role of these changes may be of importance for developing new strategies to promote neuronal survival. The objective of the present work was to evaluate neuronal survival, astroglial reaction and synaptic input changes in spinal cord anterior horn motor nuclei after ventral root avulsion in animals with EAE, both during peak disease and after remission.

**Methods:**

Lewis rats were subjected to unilateral avulsion of lumbar ventral roots (VRA) and divided into three groups: VRA control, VRA at peak of EAE, and VRA during EAE remission. The animals were sacrificed and their lumbar spinal cords processed for immunohistochemistry, transmission electron microscopy, and motoneuron counting.

**Results:**

The results indicate a reduction in astroglial reaction, a maintenance of microglial reactivity, and increases in synaptic covering of, and survival of, motoneurons in the VRA+EAE group as compared to VRA alone.

**Conclusion:**

The present findings indicate that CNS inflammation may directly influence synaptic plasticity as well as the stability of neuronal networks, positively influencing the survival of lesioned neurons.

## Background

Traumatic lesions to the spinal cord, which involve neuronal death, convey devastating and permanent loss of function. Alterations also develop in response to the consequent local hemorrhage and ischemia [1]. Among these alterations, upregulation of receptors for neurotrophic factors such as the low affinity neurotrophin receptor p75NTR [2], the high affinity trkB receptor for BDNF [3], and the GFRa-1 receptor for glial derived neurotrophic factor (GDNF) [4], are well established examples. Additionally, upregulation of proteins such as growth associated protein-43 (GAP-43) [5,6] and calcitonin gene-related peptide (CGRP), and down-regulation of receptors and enzymes involved in neurotransmission, including choline acetyltransferase (ChAT) and NMDA receptors [6], may be cited as important responses to injury that are related to a regenerative strategy [5-8] in the cell body.

In adults, such a physiological shift is more related to cell repair than to survival [8]. However, lesions close to the surface of the spinal cord may result in extensive degeneration of adult motoneurons, as is the case after avulsion of ventral roots (VRA), an experimental model used to investigate adult neuronal degeneration [6,9,10]. Koliatsos et al. [9] have demonstrated that such lesions result in retrograde death of 80% of motoneurons within the first 2 weeks. Piehl et al. [11] have shown a peak of expression of pro-inflammatory cytokines at the onset of motoneuron death, suggesting that inflammation is related to this particular type of neurodegenerative process.

Although inflammation may be harmful to the survival of lesioned neurons, the exacerbation of facial motoneuron loss after nerve transection in severe combined immunodeficient (scid) mice suggests that immune cells associated with acquired immunity play a role in regulating motoneuron survival after neuronal lesion [12]. This hypothesis is strengthened by the fact that autoimmune T cells are able to protect neurons from degeneration after axotomy within the CNS, as demonstrated by the fact that avulsed neurons survive, at a strikingly greater ratio, if the lesion is performed during the course of experimental autoimmune encephalomyelitis [4]. However, although expression of cytokines and neurotrophic factors may be involved, it is possible that synaptic plasticity events may also be influenced by the course of the disease and by the presence of T cells in nervous tissue. Synaptological changes may also be involved in neuronal degeneration. It is possible that a better understanding of the degree of input alterations in this scenario may be an important tool for developing new strategies aiming at increasing neuronal survival after proximal nerve root injuries. With this in mind, the objective of the present work was to perform a detailed analysis of synaptological changes in motoneuron synapses after VRA associated with progression of EAE.

## Methods

### Animals

Adult female Lewis rats (7 weeks old, 250 g body weight) were obtained from the Multidisciplinary Center for Biological Investigation (CEMIB/UNICAMP) and housed using a 12-hour light/dark cycle with free access to food and water. The study was approved by the Institutional Committee for Ethics in Animal Experimentation (CEUA/IB/UNICAMP, proc. n° 1494-1.), and the experiments were performed in accordance with the guidelines of the Brazilian College for Animal Experimentation. The animals were subjected to unilateral avulsion of the L4-L6 lumbar ventral roots and divided into 3 groups: Group 1 was considered a positive control, with no EAE immunization; these animals were sacrificed 2 weeks after avulsion. Groups 2 and 3 were subjected to EAE induction thirty minutes after avulsion. Animals in Group 2 were analyzed at the peak of exacerbation of EAE (degree 3) and animals in group 3 were analyzed at the remission stage of EAE, in which there is recovery from clinical signs of disease; these animals were sacrificed 2 weeks after peak disease. The animals were sacrificed and their lumbar spinal cords were processed for immunohistochemistry (n = 5 for each group), transmission electron microscopy (n = 4 for each group) and for neuronal survival counting (n = 5 for each group).

### Ventral root avulsion (VRA) and EAE induction

All reagents were obtained from Sigma-Aldrich (St, Louis, MO) unless otherwise specifically mentioned. The rats were subjected to unilateral avulsion of the lumbar ventral roots as previously described [13]. In brief, a unilateral laminectomy was performed on the L4-L6 spinal segments. The dural sac was opened by a longitudinal incision and, after dissection of the denticulate ligament, the spinal cord was gently twisted so that the ventral roots associated with lumbar enlargement were identified and avulsed. Finally, the spinal cord was replaced in its original position and the musculature, fascia and skin sutured in layers.

After lesioning, EAE was induced in groups 2 and 3. The rats were immunized with an emulsion containing guinea pig myelin basic protein (MBP, 25 μg) and complete Freund's adjuvant emulsion (CFA) supplemented with 2 mg/ml *Mycobacterium tuberculosis *H37RA (Difco Laboratories, Detroit, MI). This solution (100 μl) was injected subcutaneously in the footpad of animals' hind limbs. The animals were monitored daily, and neurological impairment was classified as follows: grade 0, no clinical signs; grade 1, tail weakness or paralysis; grade 2, hind limb paraparesis (peak disease); grade 3, hind limb paralysis (this occurred 13 days after immunization, at which time the animals in group 2 were sacrificed); grade 4, complete paralysis (tetraplegy). One group of animals (group 3) was kept alive after peak exacerbation of disease, and sacrificed 2 weeks later, after entering the remission phase.

### Specimen preparation

At the predetermined times, animals were anaesthetized with a mixture of Kensol (xylasine, Köning, Argentina, 10 mg/Kg) and Vetaset (Cetamine, Fort Dodge, USA, 50 mg/Kg) and the vascular system was rinsed by transcardial perfusion with phosphate buffer (pH 7.4). For the counting of surviving neurons and immunohistochemical detection of synaptophysin, glial fibrillary acidic protein (GFAP) and Iba1; rats were fixed by vascular perfusion with 10% formaldehyde in phosphate buffer (pH 7.4). Thereafter, the lumbar intumescence was dissected out, post-fixed overnight and then washed in phosphate buffer and stored in sucrose (20%) for 8 hours before freezing. Transverse cryostat sections (12 μm thick) of spinal cords were obtained and transferred to gelatin-coated slides, dried at room temperature for 30 min and stored at -20°C until analyzed. For electron microscopy, 100 ml of a fixative containing 2.5% glutaraldehyde and 0.5% paraformaldehyde in phosphate buffer (pH 7.4) was perfused through the ascending aorta. The lumbar spinal cord was removed and stored overnight in the same fixative at 4°C. The specimens were then trimmed and osmicated, dehydrated, and embedded in Durcupan ACM (Fluka, Steinheim, Switzerland). Ultrathin sections from the L4-L6 segments were collected on formvar-coated copper grids, contrasted with uranyl acetate and lead citrate, and examined under a Leo 906 transmission electron microscope operating at 60 kV.

### Counting of motoneurons surviving after ventral root avulsion

Cell counts were performed in double blind fashion on sections from the lumbar enlargement (*n *= 5). The animals were fixed by vascular perfusion with 10% formaldehyde in phosphate buffer (pH 7.4). The lumbar enlargement was then dissected out, post-fixed overnight in the same fixative and then frozen. Transverse cryostat sections (12 μm) of the spinal cords were obtained and transferred to silane-coated slides, which were stained for 3 minutes in aqueous 1% cresyl fast violet solution. The sections were then dehydrated and mounted with Entellan (Merck).

The motoneurons were identified based on their morphology and location in the ventral horn (dorsolateral lamina IX). Only cells with a visible nucleus and nucleolus were counted. The counts were made on 20 sections (every fourth section) along the lumbar enlargement both on the ipsilateral and contralateral sides of each spinal cord. The absolute numbers of motoneurons per section on the lesioned and non-lesioned sides were used to calculate the percentage of surviving cells in each specimen. To correct for double counting of neurons, the Abercrombie's [14] formula was used:

Where *N *is the corrected number of counted neurons, *n *is the counted number of cells, *t *is the thickness of the sections (12 μm) and *d *is the average diameter of the cells. Because differences in cell size significantly affect cell counts, the value of *d *was calculated specifically for each experimental group, for both ipsilateral and contralateral neurons. For this, the diameters of 15 randomly chosen neurons from each group were measured (ImageTool software, version 3.00, The University of Texas Health Science Center in Santo Antonio, USA) and the mean values calculated, as shown in Table 1.

**Table 1:** Motoneuron counts after avulsion and EAE induction.

	Avulsion		Avulsion + EAE-peak		Avulsion + EAE-rem	
	
	Ipsilateral	Contralateral	Ipsilateral	Contralateral	Ipsilateral	Contralateral
Number of neurons per section	1.76 ± 0.55	4.88 ± 0.98	4.64 ± 1.01	5.32 ± 0.65	3.64 ± 1.26	5.22 ± 1.03

Abercrombie's formula	0.90 ± 0.26	2.41 ± 0.44	2.39 ± 0.46	2.90 ± 0.31	2.14 ± 0.66	2.72 ± 0.48

The first row contains the absolute mean number of neurons sampled per section on the ipsilateral and contralateral sides of the spinal cord in the different experimental groups. The second row contains the mean number of surviving neurons after correction using Abercrombie's formula.

### Immunohistochemistry

**Figure 1:**
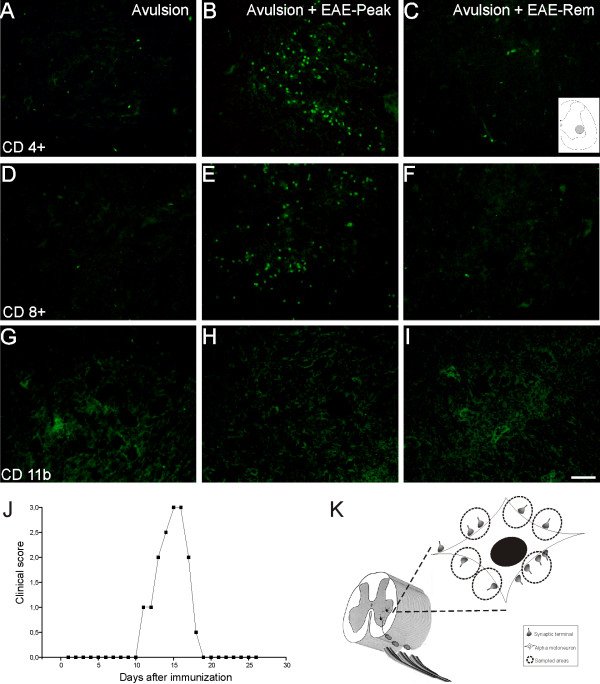
**Immune cells in spinal cord ventral horn after VRA and EAE induction**. Representative images of the sciatic nerve motor nucleus ipsilateral to the ventral root avulsion (VRA) in the studied groups. (A-C) CD4^+ ^T cell immunolabeling. (D-F) CD8^+ ^T cell immunolabeling. (G-I) CD11b-positive cell immunolabeling. A, D and G are ipsilateral to the VRA. B, E and H are ipsilateral to the VRA+EAE at peak disease. Observe the increased presence of T cells. C, F and I are ipsilateral to VRA+EAE during remission. (J) Graph representing the evolution of EAE in Lewis rats after immunization with MBP. (K) Schematic representation of sciatic nerve motoneurons in ventral horn of spinal cord after ventral root avulsion. One motoneuron is shown in detail with apposed presynaptic terminals. The dashed circles represent areas where measurements of the integrated density of pixels were performed. Scale bar = 50 μm.

Transverse sections of spinal cord (12 μm) were cut (*n *= 5) in a cryostat (Microm) and incubated with the following primary antibodies: rabbit anti-synaptophysin (Dako, 1:100), goat anti-GFAP (Chemicon, 1:200), rabbit anti-Iba1 (Wako, 1:1400), mouse anti-CD4+ (Serotec, 1:100), mouse anti-CD8+ (Serotec, 1:100) and rat anti-CD11b (BD Pharmingen, 1:100). With regard to specificity, the anti-human synaptophysin antibody reacts with a single band of 38 kDa on immunoblotting, corresponding to the protein P38 as described previously [15,16]. The immunolabeling pattern observed here was similar to that in other studies of mouse neurons, providing a characteristic punctate labeling [17,18]. The antibody against glial fibrillary acidic protein (GFAP), which is an intermediate filament (IF) protein belonging to the type III subclass of IF proteins, reacts with a single band of 52 kDa on immunoblotting [19]. The antibody used here showed a typical immunostaing pattern for astroglial cells, comparable to that described previously for mouse brain [20]. The Iba1 (a calcium-binding protein) antiserum has been previously characterized in rat brain as a marker for microglia by comparing it with staining for OX42 and B4-isolectin from *Griffonia simplicifolia*, all of which show typical microglial staining patterns [21]. CD4 protein is expressed on membrane surfaces of CD4^+ ^T cells (helper), while CD8^+ ^T cells (cytotoxic) express CD8 glycoprotein on their surfaces. CD11b is expressed by mature monocytes, natural killer cells, and a subset of lymphocytes. Sections were incubated overnight in a moist chamber at 4°C. The primary antisera were diluted in a solution containing BSA and Triton X in 0.01 M PBS. After rinsing, the secondary antibodies were applied and incubated for 45 minutes, according to the primary antibody hosts (CY-2 and CY-3, Jackson Immunoresearch; 1:250). The sections were then rinsed in PBS, mounted in a mixture of glycerol/PBS (3:1), and examined using a fluorescence microscope (TS-100, Nikon, Tokyo, Japan) equipped with a CCD camera (DMX1200, Nikon). For quantitative measurements, 3 representative images of the ipsi- and contralateral ventral horn were captured from each animal for all experimental groups, totaling 15 sampled images from each side per group. Quantification was performed with the enhance contrast and density slicing features of IMAGEJ software (version 1.33u, National Institutes of Health, USA). The integrated density of pixels (sum of the gray values of each pixel in a determined area) around each motoneuron identified in the lateral motor nucleus was measured in six circular areas of 100 μm^2 ^from each side, as shown in Figure 1K. This quantification method measures the intensity of fluorescence in a given image, and has been used in previous studies [22-25]. For synaptophysin immunolabeling, a lesioned/unlesioned ratio for the integrated density of pixels was calculated for each section (axotomy groups) and then as a mean value for each spinal cord. The data are represented as mean ± SE.

### Analysis of the ultrathin sections

Neurons with large cell bodies (35 μm in diameter), found in the sciatic motoneuron pool and cut in the nuclear plane, were identified as alpha motoneurons by the presence of C-type nerve terminals. The surfaces of these cells were then sequentially digitized at a magnification of 16,400× using a video camera connected to a computerized system, plus the acquisition feature of the Kontron KS300 software (Zeiss, Jena, Germany). The images were then mounted together in vectorial software, and the total perimeter of the neurons (in micrometers) was measured. Synaptic terminals apposing the motoneuron somata were identified, and their numbers per 100 μm of cell membrane and length of apposition as a percentage of membrane length were calculated using the measurement tool of the image tool software (version 3.0; The University of Texas Health Center in Santo Antonio). The terminals were typed under high magnification (at least 25,000×) as F (with flattened synaptic vesicles, inhibitory inputs), S (with spherical synaptic vesicles, excitatory inputs), or C (cholinergic inputs), according to the nomenclature of Conradi [26]. The distance between consecutive nerve terminals covering the motoneurons was also determined. The data are represented as mean ± SE. In total, three neurons per animal in each of 3 groups of four animals were examined (n = 12 neurons for each of 3 groups: VRA, VRA at peak of EAE and VRA during EAE remission).

### Statistical analysis

The data were analyzed by ANOVA and two-tailed Student's *t*-test for parametric data, or by a two-tailed Mann-Whitney U test for nonparametric data. *p *< 0.05 (*), and values with *p *< 0.01 (**) were considered statistically significant.

## Results

### EAE increases T cell influx

In order to monitor the presence of immune cells in the spinal cord microenvironment during the peak of EAE, immunohistochemistry for CD4+ and CD8+ lymphocytes, as well as CD11b+ macrophages, was performed. As seen in Figure 1, during peak disease a significant accumulation of T cells occurs, with a predominance of CD4+ T cells. In contrast, during remission and after avulsion alone, such influx of T cells is diminished or absent. Note the macrophage immunoreactivity in all studied groups.

### EAE influences neuronal survival after VRA

To quantify the effect of acute immune cell influx on survival of motoneurons, numbers of motoneurons were counted on lesioned and unlesioned sides. The raw data were subjected to Abercrombie's formula in order to avoid double counting (Table 1).

**Figure 2:**
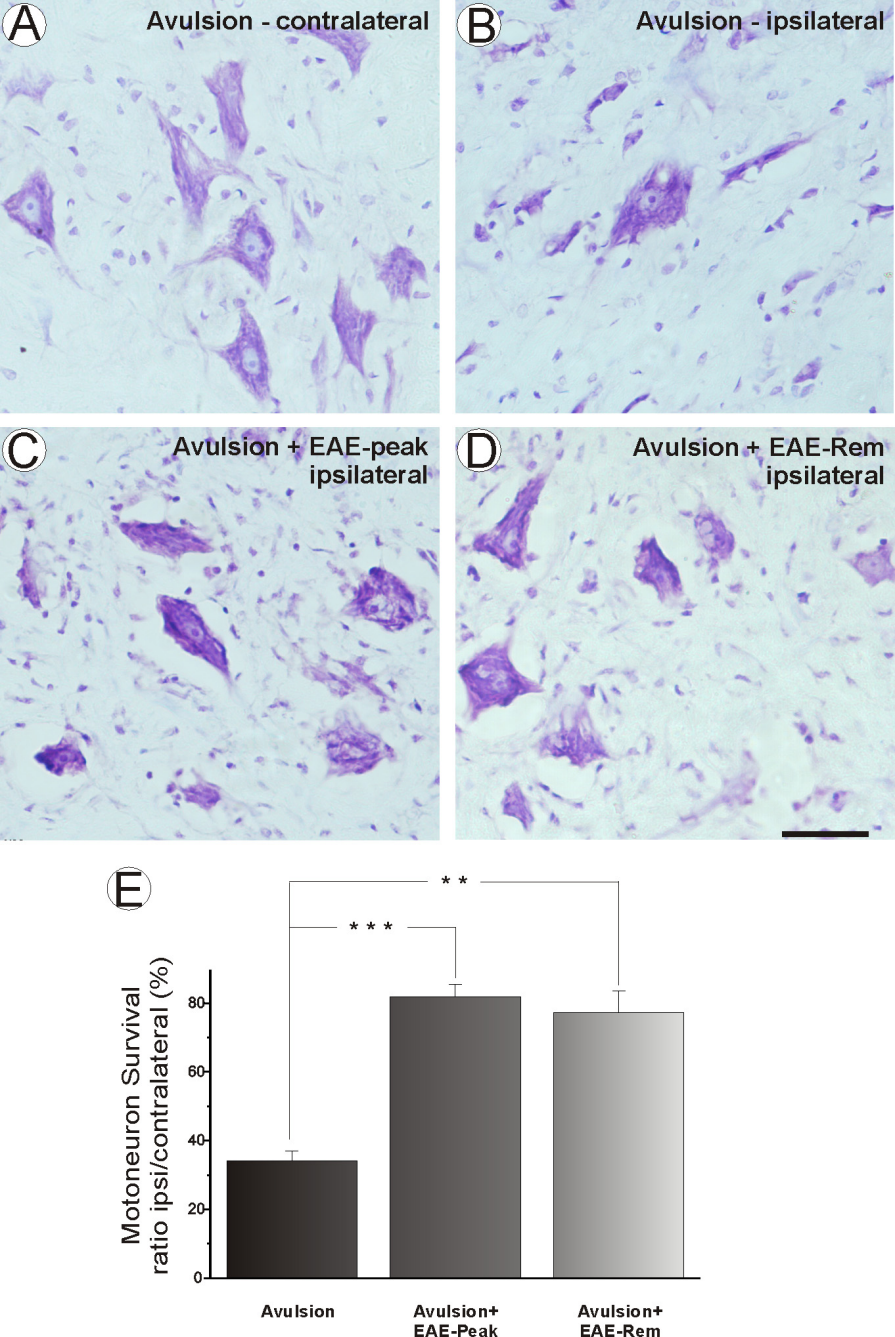
**Motoneuron survival after avulsion is increased in EAE-induced groups**. Motoneuron cell bodies of the normal (contralateral to lesion) side (A), VRA, ipsilateral side (B), VRA+EAE at peak disease, ipsilateral side (C), AVR+EAE-remission phase, ipsilateral side (D). Percentage of spinal motoneurons surviving after ventral root avulsion and immunization (E). Note a significant rescue of lesioned neurons in the EAE-induced groups (* = p < 0.05). Scale bar = 50 μm.

Figure 2 represents the percentage of surviving neurons in the different experimental groups. A neuroprotective effect of inflammation is present, which was statistically significant at p < 0.05, reflecting an approximately 50% increase in neuronal survival (VRA 34.12% ± 6.58; VRA+EAE-peak 81.87% ± 8.03; VRA+EAE-rem 77.37% ± 13.72 mean ratio ipsilateral/contralateral ± SE). The absolute number of surviving motoneurons is presented in Table 1.

### Reduction of synaptic elimination and decreased astroglial reaction after inflammation and ventral root avulsion (Figures 3, 4 and 5)

**Figure 3:**
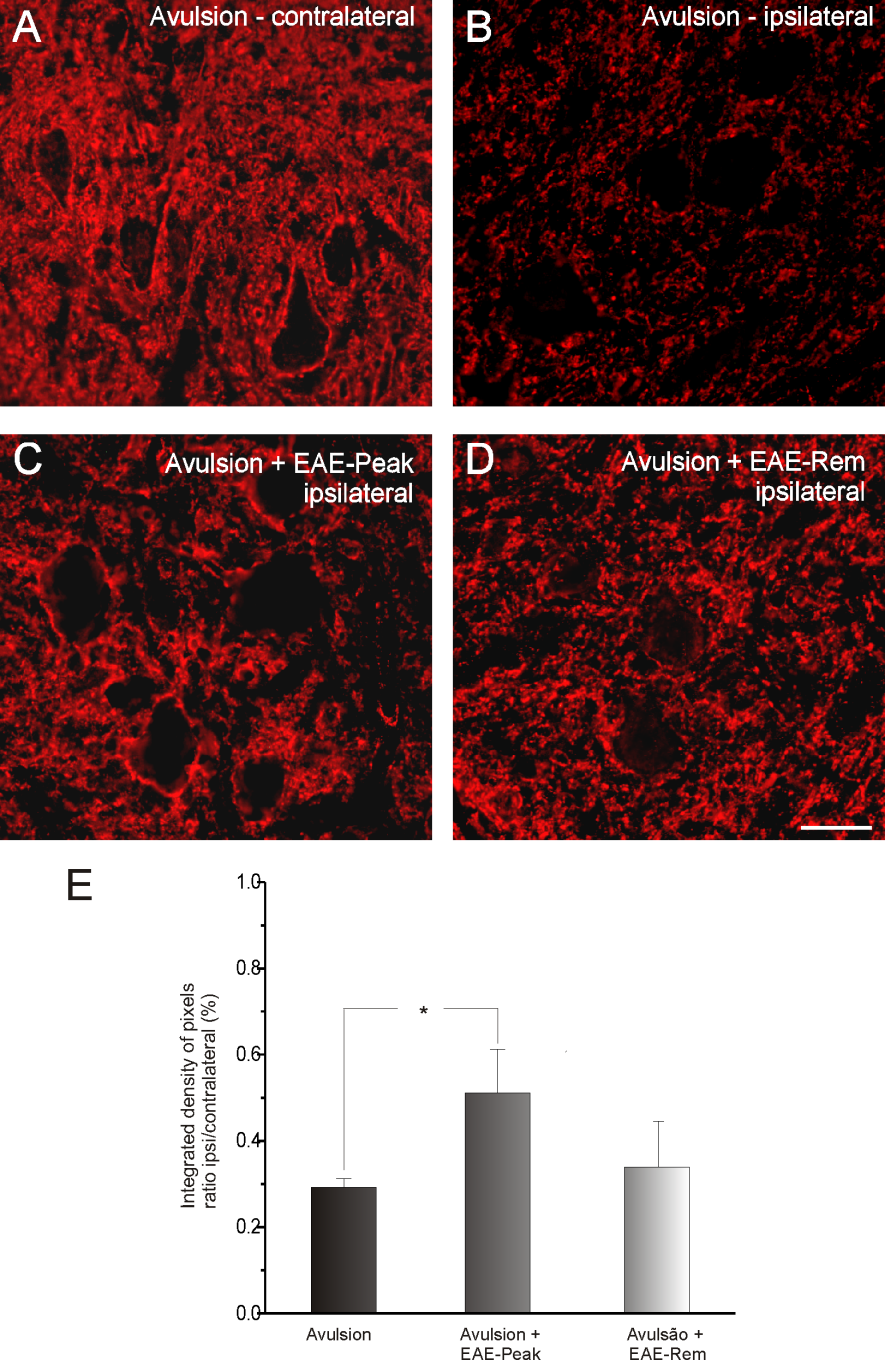
**Synaptophysin immunolabeling in spinal cord ventral horn**. Normal synaptophysin immunoreactivity on the side contralateral to the lesion (A). Observe the less intense labeling in the ipsilateral side after the avulsion alone (B); synaptic labeling is greater if avulsion is combined with EAE (C and D). (E) Graph representing quantification of immunolabeling for all groups. (* = p < 0.05). Scale bar = 50 μm.

**Figure 4:**
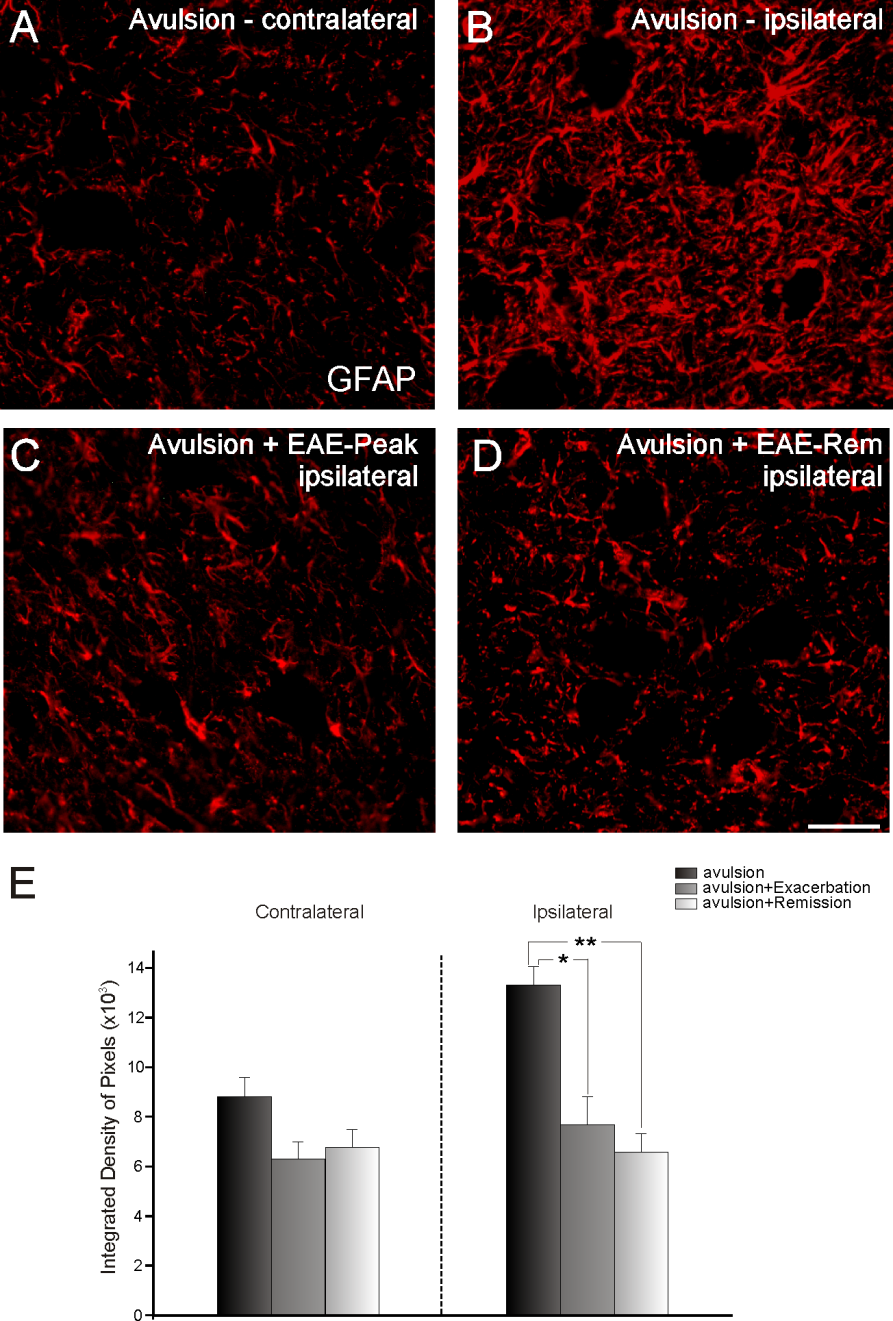
**Glial fibrillary acidic protein (GFAP) immunolabeling in spinal cord ventral horn**. Normal GFAP immunolabeling on the side contralateral to the lesion (A). Observe the increase in labeling on the ipsilateral side after avulsion alone (B). The combination of VRA and EAE (C and D) resulted in decreased astroglial reaction. (E) Graph representing quantification of immunolabeling for all groups (ipsi and contralateral sides shown separately). (** = p < 0.01). Scale bar = 50 μm.

**Figure 5:**
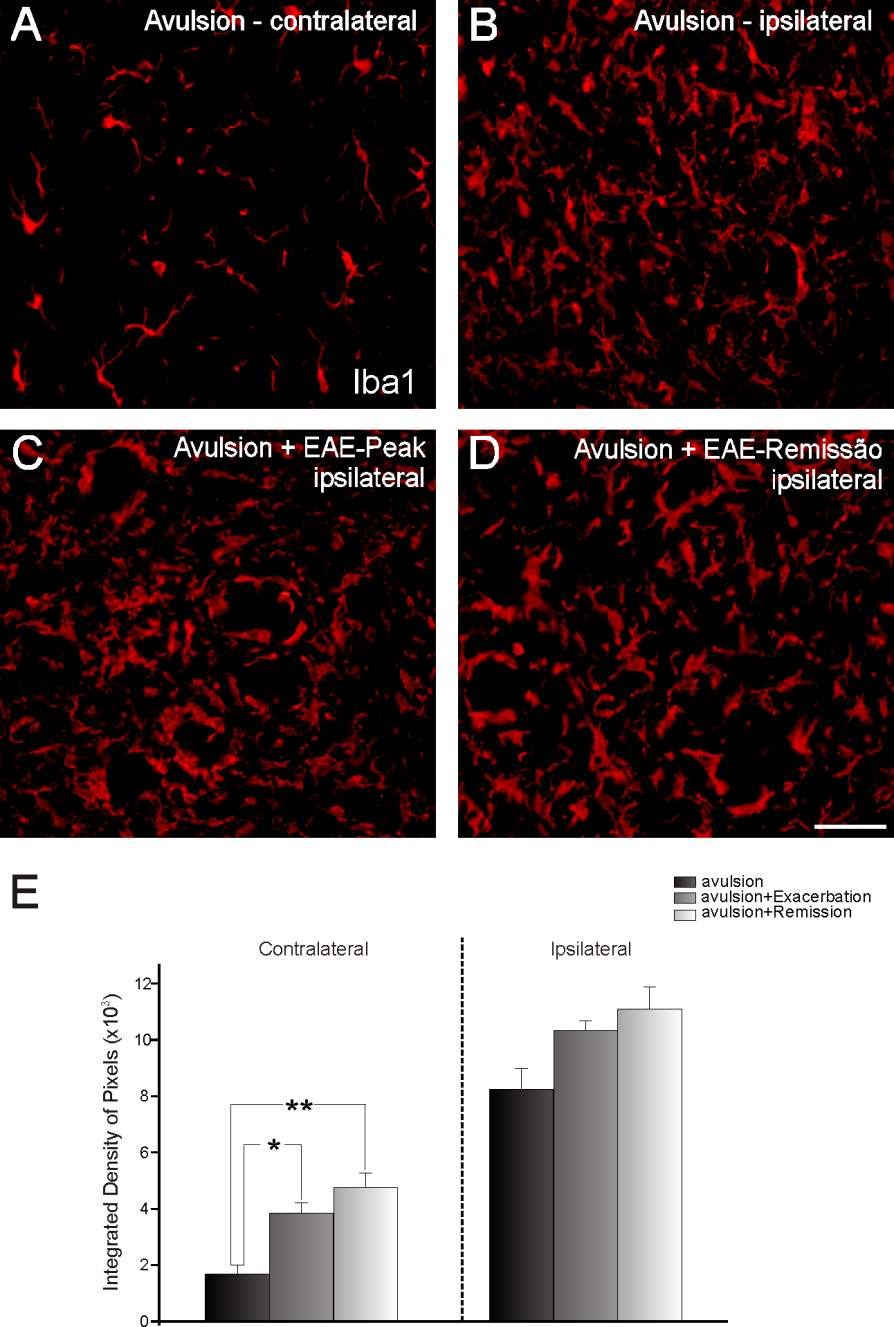
**Iba1 immunolabeling in spinal cord ventral horn**. Normal immunolabeling for Iba1 on the side contralateral to the lesion (A). Avulsed side (B) showing a marked increase in Iba-1 labeling, which was comparable in both lesioned and EAE-induced groups (C and D). (E) Graph representing quantification of immunolabeling for all groups. (ipsi and contralateral sides shown separately) (* = p < 0.05). Scale bar = 50 μm.

In order to evaluate changes in VRA synaptophysin labeling after EAE, we performed quantitative measurements of synaptophysin immunoreactivity in sciatic motor nuclei of animals after avulsion alone, or after avulsion plus EAE induction at either exacerbation or remission stages. As shown in Figure 3, VRA alone led to a significant decrease in synaptophysin expression. In contrast, VRA plus EAE at exacerbation resulted in preservation of synaptophysin immunoreactivity, especially in the immediate vicinity of motoneurons, indicating a decreased loss of inputs, putatively in apposition to the alpha motoneurons. These results show that the preservation of inputs after avulsion combined with exacerbation of EAE is statistically different from VRA alone (VRA - 0.29 ± 0.02; VRA+EAE-peak - 0.51 ± 0.11; VRA+EAE-rem - 0.34 ± 0.06; mean ratio ipsilateral/contralateral ± SE).

Immunoreactivity against GFAP was used to analyze the degree of astroglial reactivity after avulsion and immunization (Figure 4). Figure 4A shows GFAP immunoreactivity in the spinal cord contralateral to the lesion in avulsed animals without EAE. This demonstrates the presence of GFAP-positive astrocytic processes in the microenvironment close to large motoneurons. There was no additional GFAP immunolabeling in the contralateral spinal cord of animals with avulsion lesions + EAE (VRA - 8.82 × 10^3 ^± 7.5 × 10^2^; VRA+EAE-peak - 6.32 × 10^3 ^± 6.6 × 10^2^; VRA+EAE-rem - 6.78 × 10^3 ^± 7.8 × 10^2^; integrated density of pixels ± SE; Figure 4E). On the side ipsilateral to lesioning, there was a significant increase in astrocyte reactivity after ventral root avulsion as demonstrated by the presence of reactive gliosis in the affected segments of the spinal cord. Increased GFAP labeling was particularly concentrated in the vicinity of the avulsed motoneurons, suggesting a possible communication between lesioned neurons and glia (Figure 4B). However, the association of VRA with EAE resulted in less intense astrogliosis in the ipsilateral lesioned side both at peak disease and during remission (VRA - 13.32 × 10^3 ^± 7.4 × 10^2^; VRA+EAE-peak - 7.70 × 10^3 ^± 9.8 × 10^2^; VRA+EAE-rem - 6.58 × 10^3 ^± 7.3 × 10^2^; integrated density of pixels ± SE).

In contrast to the GFAP results, Iba1 immunolabeling in the contralateral sides of the three experimental groups showed increases in microglial reactivity with the association of EAE+VRA (VRA - 1.69 × 10^3 ^± 3.1 × 10^2^; VRA+EAE-peak - 3.86 × 10^3 ^± 3.6 × 10^2^; VRA+EAE-rem - 4.76 × 10^3 ^± 5.1 × 10^2^; integrated density of pixels ± SE; Figure 5E). However, the increases in microglial reactivity were not statistically different from the control group in the ipsilateral analysis, indicating that the combination of EAE+VRA did not result in further activation of microglial cells (VRA - 8.25 × 10^3 ^± 7.4 × 10^2^; VRA+EAE-peak - 10.34 × 10^3 ^± 3.4 × 10^2^; VRA+EAE-rem - 11.11 × 10^3 ^± 7.8 × 10^2^; integrated density of pixels ± SE).

### VRA decreases synaptic elimination during the induction phase of EAE

**Figure 6:**
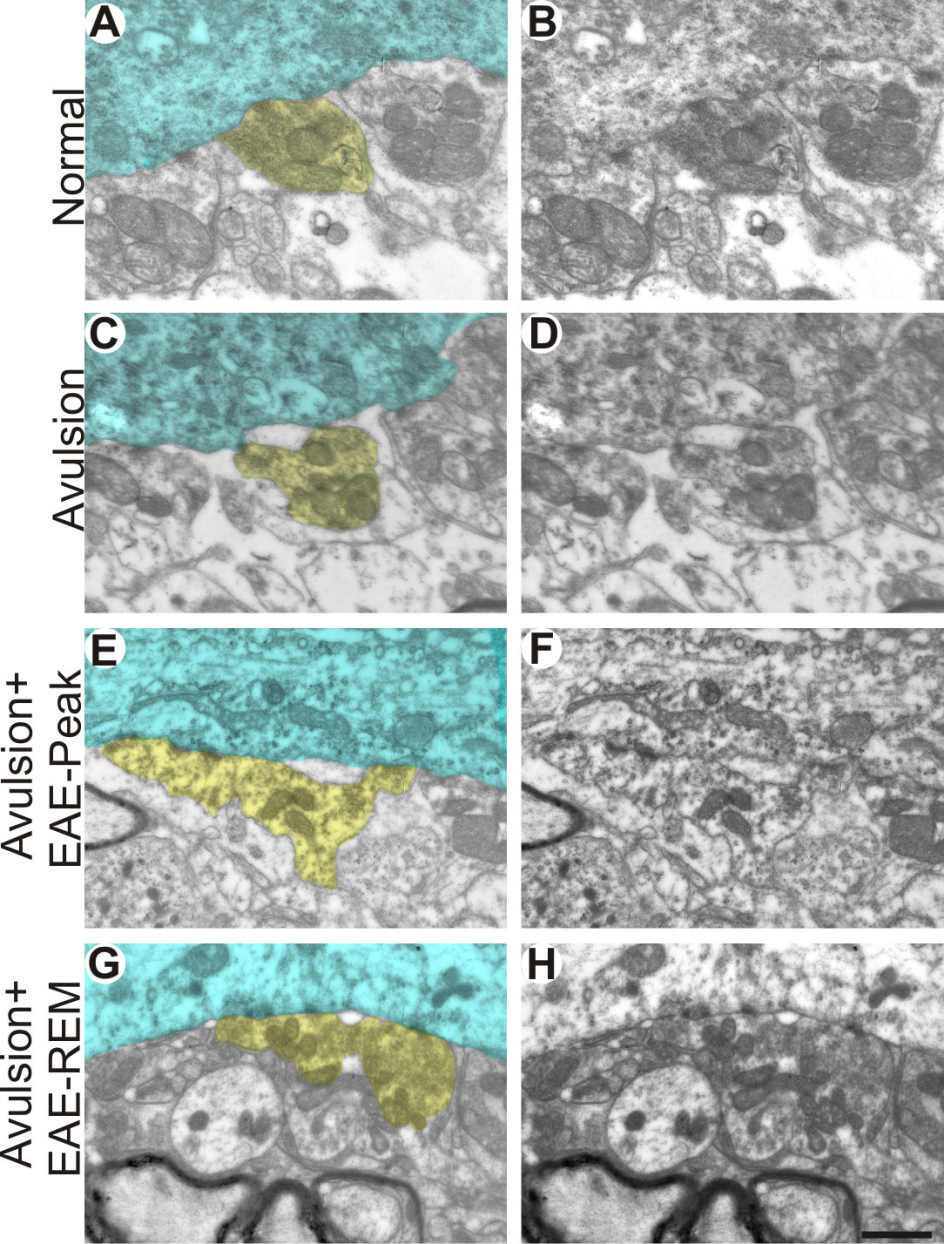
**Ultrastructure of synapses apposed to alpha motoneurons**. Normal input coverings of the surface of an alpha motoneuron (A-B). Synaptic covering with partially detached terminals intermingled with astrocytes in VRA alone (C and D), VRA-EAE-peak (E and F) and VRA-EAE-rem groups (G and H). In A, C, E and G, one of the terminals is highlighted in yellow and the motoneuron cytoplasm is colorized in cyan. Scale bar = 1 μm.

**Figure 7:**
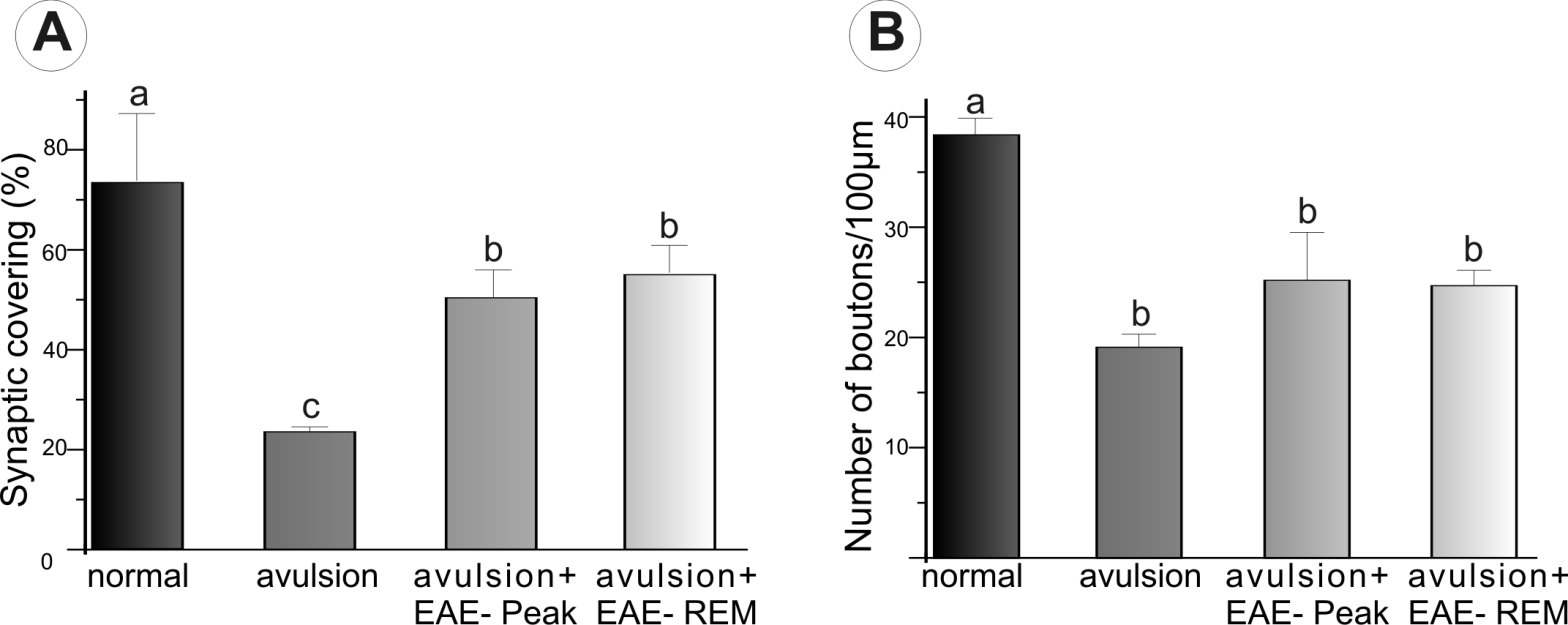
**Ultrastructural analysis of alpha motoneuron synaptic coverings**. Quantitative ultrastructural analysis of synaptic coverings and number of boutons/100 μm of motoneuron membrane. (A) Percentage reduction in synaptic covering in the experimental groups after injury as well as a protective effect of inflammation. (B) Number of boutons/100 μm of membrane; there were no statistically significant differences between the lesioned groups. Bars marked with different letters are statistically different at p < 0.05.

A series of synaptic changes were identified at the ultrastructural level after VRA through a detailed analysis of synaptic coverings of, as well as of numbers of inputs in apposition to, large motoneurons. Figure 6 shows normal neurons on the contralateral side of VRA-only spinal cords, and the process of synaptic retraction (on the ipsilateral side) that occurs following VRA. Such changes were most evident in the avulsed-alone group, with a 68% decrease in overall synaptic covering of ipsilateral, compared to contralateral, neurons (Figure 7) (normal contralateral neurons - 73.40 ± 13.54; ipsilateral neurons after VRA - 23.65 ± 0.05; VRA + EAE-peak - 50.47 ± 5.06; VRA+EAE-rem - 55.11 ± 5.41; mean ratio ± SD). The number of inputs/100 μm in apposition to the neuronal membranes after avulsion only was lower when compared to other groups. However, there was no statistical difference when comparing the avulsion alone to the immunized groups (contralateral side - 39.49 ± 3.37; VRA - 19.22 ± 1.14; VRA+EAE-peak - 25.5 ± 3.92; VRA+EAE-rem - 24.73 ± 1.37 mean ratio ± SE).

**Figure 8:**
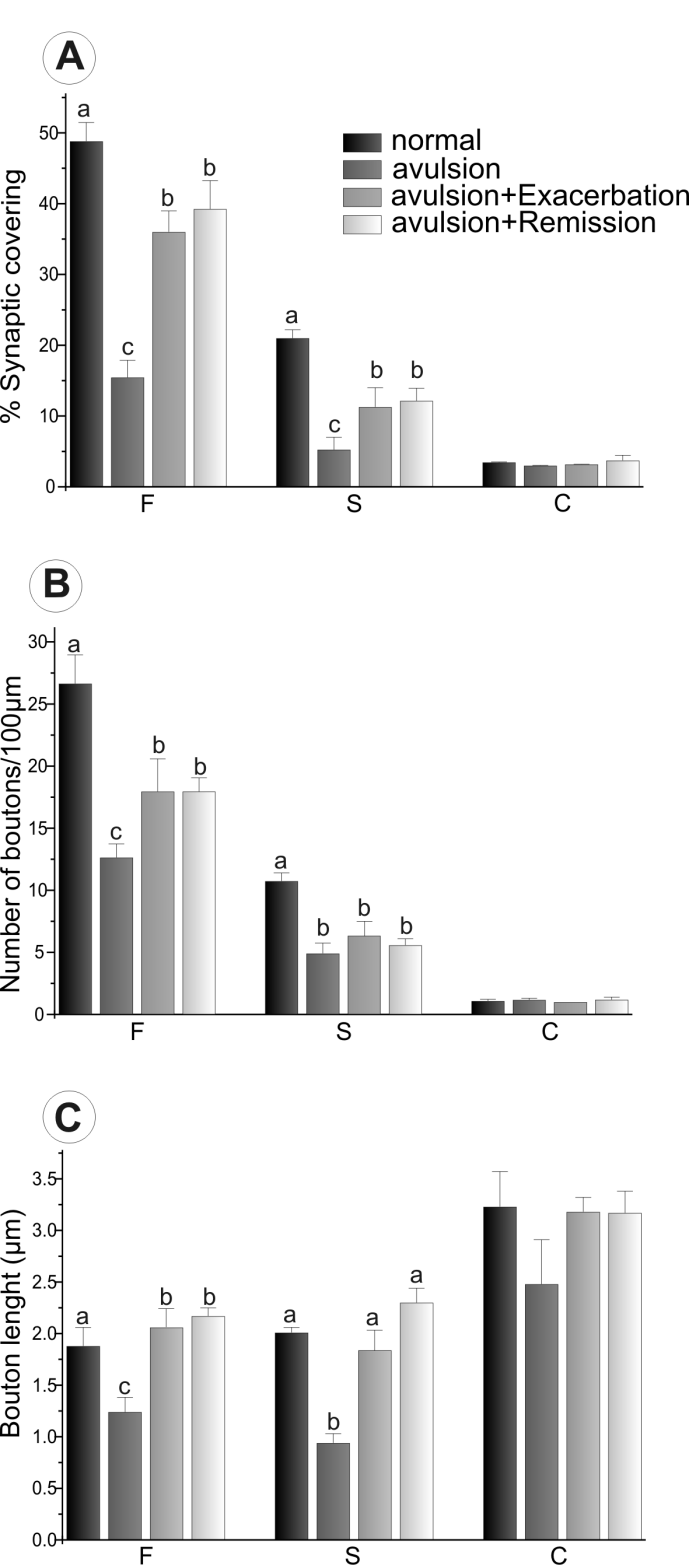
**Synaptic terminal analysis based on the morphology of synaptic vesicles**. Detailed quantitative analysis of F, S and C terminals. (A) Synaptic coverings, (B) Number of boutons/100 μm of motoneuron membrane and (C) Bouton length. Bars marked with different letters are statistically different at p < 0.05.

Synapse types (F-terminals, representing inhibitory inputs; S-terminals, representing excitatory inputs; and C-terminals, representing cholinergic inputs) in apposition to the cell bodies were also studied (Figure 8). In this regard, there was a more prominent loss of both excitatory (S terminals) and inhibitory inputs after VRA only as compared to the other groups.

### Patterns of terminal distribution after induction of EAE

**Figure 9:**
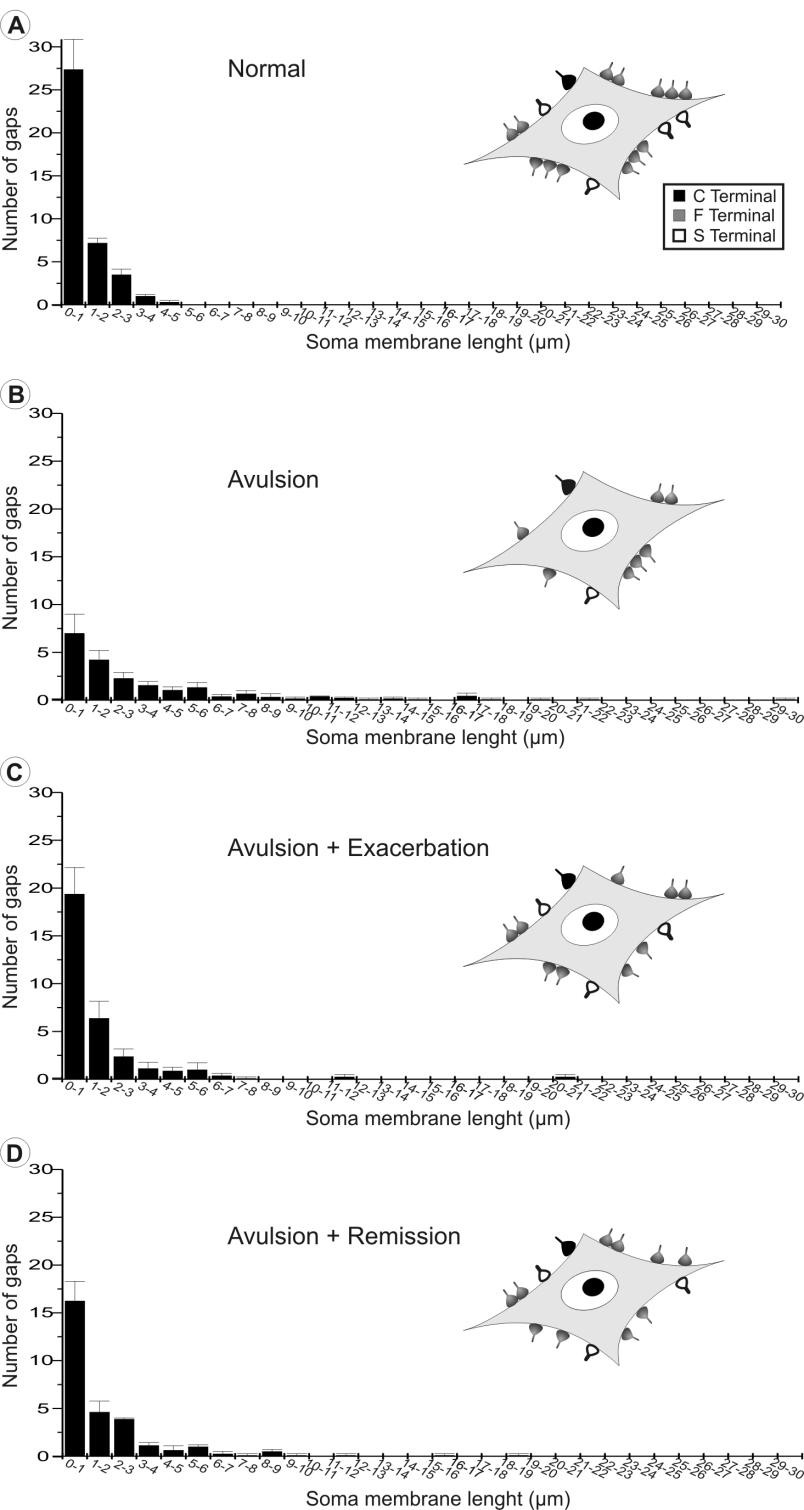
**Clustering of terminals on the surfaces of motoneurons after VRA and EAE induction**. Distribution of gap lengths between clusters of terminals apposing motoneuron soma cell membranes. (A) Normal distribution of intervals between nerve terminals showing that boutons are organized in clusters of inputs. (B) Distribution in VRA-only animals showing alteration by extensive retraction of terminals. (C and D) Decreased presence of large gaps between terminals after the combination of EAE and VRA.

Figure 9 represents the pattern of synaptic terminal distributions along motoneuron surfaces in the different experimental groups. In normal neurons (on the contralateral side of VRA-only spinal cords) the interval between terminals was usually short, from 1 to 3 μm, so that clusters of synapses could be identified. However, after VRA alone, the size of such intervals on the ipsilateral side increased up to 29 μm, resulting in a decreased frequency of short gaps. The immunized groups showed an intermediate pattern demonstrating partial retention of input clusters.

## Discussion

The present work indicates that active EAE may have a neuroprotective potential, demonstrable after a severe proximal nerve root lesion, namely ventral root avulsion (VRA). It has been shown that only a few T cells are recruited into the lesion site after this type of lesion [7]. In contrast, the course of EAE induces a substantial influx of CD4+ and CD8+ T cells to the spinal cord microenvironment, resulting in local upregulation of neurotrophic factors and cytokines. Such T lymphocyte migration into the CNS after lesioning or during the course of an autoimmune disease such as the EAE may play a role in regulating motoneuron survival [27,28].

The neuroprotective role of inflammation has been addressed in some instances in the literature, and the presence of activated T cells and the subsequent release of cytokines and neurotrophic factors is thought to directly influence neuronal responses to injury. In fact, Serpe et al. [12] have shown a neuroprotective role for inflammation after injury resulting in increased survival of motoneurons following transection of the facial nerve. In this regard, involvement of the potent neurotrophic factor BDNF and two anti-inflammatory cytokines, namely IL-10 and transforming growth factor, have been demonstrated previously [29,30]. This is in line with the work of Hammarberg et al. [4], who described rescue of a significant number of avulsed motoneurons in animals receiving MBP immunization leading to a monophasic form of EAE. Such neuroprotection was associated with the presence of elevated levels of NT-3, BDNF and GDNF, possibly produced by T and NK cells.

Our immunohistochemical data, as well as counts of motoneurons after VRA+EAE and avulsion alone, are in agreement with previous reports, and indicate that such a proximal nerve root lesion leads to a significant decrease of motoneuron-associated synaptic activity as manifested by decreased synaptophysin immunoreactivity. Also, both astrocytes and microglia became significantly reactive. The induction of EAE in VRA-lesioned animals resulted in partial preservation of synaptophysin labeling and rescue of lesioned motoneurons, indicating a neuroprotective role for T cell influx into the spinal cord. Also, this inflammatory process may have influenced glial responses to injury. This was particularly strong for astroglial reactions, which were milder when VRA was associated with EAE. Microglial cells, on the other hand, showed increased reactivity on the side ipsilateral to the lesion, but these changes were not statistically significant, indicating that the association of VRA+EAE did not significantly contribute to further activation of microglial cells.

The immunolabeling findings described herein are in agreement our ultrastructural findings. Ultrastructural analysis has not been, to our knowledge, performed after the combination of VRA and exacerbation of EAE. Thus the present work aimed to give a detailed analysis of the synaptological changes induced by the evolution of EAE when associated with a proximal lesion that induces motoneuron degeneration. Compared to the contralateral side, there was synaptic loss on the side ipsilateral to VRA lesioning in the EAE-induced group, which is in agreement with previous findings [31-33]. However, the present results indicate that the combination of EAE and VRA results in statically significant preservation of total synaptic coverings of motoneurons, in comparison to avulsion alone. To better understand whether there is a qualitative difference in pattern of synaptic detachment between the AVR+EAE and avulsion-alone situations, the fate of various types of synapses was investigated, in a manner similar to described by Oliveira et al. [34]. Classification of synaptic terminals was based on the shape of vesicles within synaptic boutons, according to Conradi [26]. Interestingly, the greater synaptic covering found in VRA+EAE material was mainly associated with F terminals, which are more numerous than S inputs. Preferential elimination of glutamatergic inputs after a lesion to the spinal cord has been described previously [35]. In that study, a longitudinal incision in the ventral funiculus of the spinal cord, thus cutting motor axons very proximally, led to an extensive retraction of presynaptic terminals in apposition to the axotomized motoneurons. Such an input elimination is possibly controlled by the motoneuron itself, so that terminals using glutamate as neurotransmitter are disconnected to a larger extent than nerve terminals with inhibitory amino acids. This indicates that lesioned neurons avoid a process of excitotoxicity that could ultimately result in cell loss. In this way, it is possible that the upregulation of trophic factors described earlier by others may have a positive effect on motoneurons afferents, resulting in a greater survival ratio.

## Conclusions

The present findings indicate that CNS inflammation may directly influence synaptic plasticity as well as stability of neuronal networks, increasing survival of lesioned neurons. Such events are possibly linked to the influx of lymphocytes and the production of various neurotrophic molecules that is associated with peak exacerbation of EAE.

## List of abbreviations

VRA: Avulsion of lumbar ventral roots; BDNF: Brain derived neurotrophic factor; CGRP: Calcitonin gene-related peptide; CNS: Central nervous system; ChAT: Choline acetyltransferase; GABA: Gamma-aminobutyric acid; MBP: Guinea pig myelin basic protein; GDNF: Glial derived neurotrophic factor; EAE: Experimental autoimmune encephalomyelitis; GFAP: Glial fibrillary acidic protein; EAE-peak: Peak of EAE; EAE-rem: Remission of EAE.

## Competing interests

The authors declare that they have no competing interests.

## Authors' contributions

ALRO provided the study concept, design and supervision. RB participated on the experimental design and acquisition of data. Both authors provided analysis and interpretation and participated in drafting of the manuscript. Both authors read and approved the final manuscript.
